# The Novel benzamide Derivative, VKNG-2, Restores the Efficacy of Chemotherapeutic Drugs in Colon Cancer Cell Lines by Inhibiting the ABCG2 Transporter

**DOI:** 10.3390/ijms22052463

**Published:** 2021-02-28

**Authors:** Silpa Narayanan, Nehaben A. Gujarati, Jing-Quan Wang, Zhuo-Xun Wu, Jagadish Koya, Qingbin Cui, Vijaya L. Korlipara, Charles R. Ashby, Zhe-Sheng Chen

**Affiliations:** 1 Department of Pharmaceutical Sciences, College of Pharmacy and Health Sciences, St. John’s University, Queens, NY 11439, USA; silpa.narayanan16@my.stjohns.edu (S.N.); nehagujarati@gmail.com (N.A.G.); jingquan.wang16@my.stjohns.edu (J.-Q.W.); zhuoxun.wu17@my.stjohns.edu (Z.-X.W.); jagadish.koya16@my.stjohns.edu (J.K.); qingbc@gmail.com (Q.C.); KORLIPAV@stjohns.edu (V.L.K.); cnsratdoc@optonline.net (C.R.A.J.); 2 School of Pharmaceutical Science, Guangzhou Medical University, Guangzhou 511436, China

**Keywords:** benzamide, MDR, reversal effect, ATP binding cassette, ABCG2

## Abstract

The overexpression of ATP-binding cassette transporter, ABCG2, plays an important role in mediating multidrug resistance (MDR) in certain types of cancer cells. ABCG2-mediated MDR can significantly attenuate or abrogate the efficacy of anticancer drugs by increasing their efflux from cancer cells. In this study, we determined the efficacy of the novel benzamide derivative, VKNG-2, to overcome MDR due to the overexpression of the ABCG2 transporter in the colon cancer cell line, S1-M1-80. *In vitro*, 5 μM of VKNG-2 reversed the resistance of S1-M1-80 cell line to mitoxantrone (70-fold increase in efficacy) or SN-38 (112-fold increase in efficacy). In contrast, *in vitro,* 5 μM of VKNG-2 did not significantly alter either the expression of ABCG2, AKT, and PI3K p110β protein or the subcellular localization of the ABCG2 protein compared to colon cancer cells incubated with the vehicle. Molecular docking data indicated that VKNG-2 had a high docking score (−10.2 kcal/mol) for the ABCG2 transporter substrate-drug binding site whereas it had a low affinity on ABCB1 and ABCC1 transporters. Finally, VKNG-2 produced a significant concentration-dependent increase in ATPase activity (EC_50_ = 2.3 µM). In conclusion, our study suggests that *in vitro*, VKNG-2 reverses the resistance of S1-M1-80, a cancer cell line resistant to mitoxantrone and SN-38, by inhibiting the efflux function of the ABCG2 transporter.

## 1. Introduction 

**Scheme 1:**
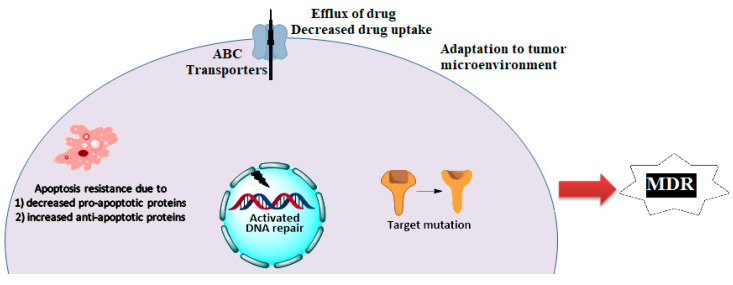
Mechanisms of Multidrug resistance in cancer.

Cancer chemotherapy has made significant advances in decreasing the mortality and improving the quality of life for cancer patients [1,2,3]. Nonetheless, cancer is the second leading cause of death and globally, causing an estimated 9.6 million deaths in 2018 [4]. Despite the availability of numerous chemotherapeutic drugs, the 5-year survival rates for many cancers are low due to resistance to these drugs produced by innate or acquired mechanisms [5,6,7,8,9]. Multidrug resistance (MDR) is among the primary causes for the failure of anticancer efficacy and the high mortality rates in metastatic cancer patients [2,7,10,11]. MDR can be defined as the acquisition of resistance to numerous structurally unrelated antineoplastic drugs in cancer cells or tumors [10,11,12]. The various factors that mediate MDR are shown in Scheme 1.

Globally, colorectal cancer (CRC) has been reported to be the third most common cancer and the fourth most typical cause of cancer-associated deaths [13]. Current approaches for treating CRC are listed in Table 1. Furthermore, the use of monoclonal antibodies has been reported to increase progression-free survival (PFS) in CRC patients [14]. The majority of the above-mentioned regimens have improved the response rate, PFS and overall survival rates in CRC patients. However, the long - term management of CRC has been relatively poor due to acquired drug resistance and the presence of certain mutations in colorectal cancer tumors [14,15,16].

The overexpression of certain ATP binding cassette (ABC) transporters can mediate the resistance of cancer cells to various anticancer drugs [11,17,18]. The ABC transporter superfamily consists of a total of 48 members that are subdivided into subfamilies from ABCA to ABCG [17,19,20]. The ABCG2 transporter, also known as the breast cancer resistant protein (BCRP), is expressed in the placenta, blood-brain barrier, liver, kidney, testis, colon epithelium, and gastrointestinal tract and it plays a protective role by extruding xenobiotics and other drugs, as well as endogenous molecules [21,22]. It contains a transmembrane binding domain and a nucleotide-binding domain and is activated upon homodimerization [23]. Examples of the substrates for the ABCG2 transporter are listed in Table 1. ABCG2 also plays a protective role by attenuating oxidative stress-induced cell damage and death in the colorectal cancer cell line, HT-29 [24].

Numerous *in vitro* studies have reported the presence of polymorphisms in ABC transporter proteins, such as ABCC1, ABCC2, and ABCG2, which mediate the discrepancy in drug toxicity between patients and drug resistance to irinotecan and SN-38 in patients with CRC [25].

*In vitro* data indicate that the overexpression of the ABCG2 transporter by HCT-116 colon cancer cells produces resistance to SN-38 (an active metabolite of irinotecan), which is a substrate for the ABCG2 transporter [26]. Hsu et al. (2018) reported that the ABCG2 transporter decreases oxaliplatin-induced apoptosis in LoVo colon cancer cells [27]. Various benzamide compounds are efficacious in inhibiting the efflux activity of ABCG2 [28]. Kathawala et al. [28] reported that the compound, 2-trifluoromethyl-2-hydroxypropionamide, a benzamide derivative, decreased ABCG2-mediated resistance by inhibiting the efflux activity of ABCG, and increased the efficacy of mitoxantrone and SN-38 in NCI-H460/MX20 cancer cells. Thus, in this study, we have conducted experiments to determine (1) if our novel compound, VKNG-2, a benzamide derivative with a urea linker, is efficacious in reversing MDR in the drug-resistant S1-M1-80 cells overexpressing the ABCG2 transporter and (2) the mechanism of action of VKNG-2.

## 2. Results

### 2.1. VKNG-2 Sensitizes ABCG2 Overexpressing Cells to Mitoxantrone and SN-38

**Figure 1:**
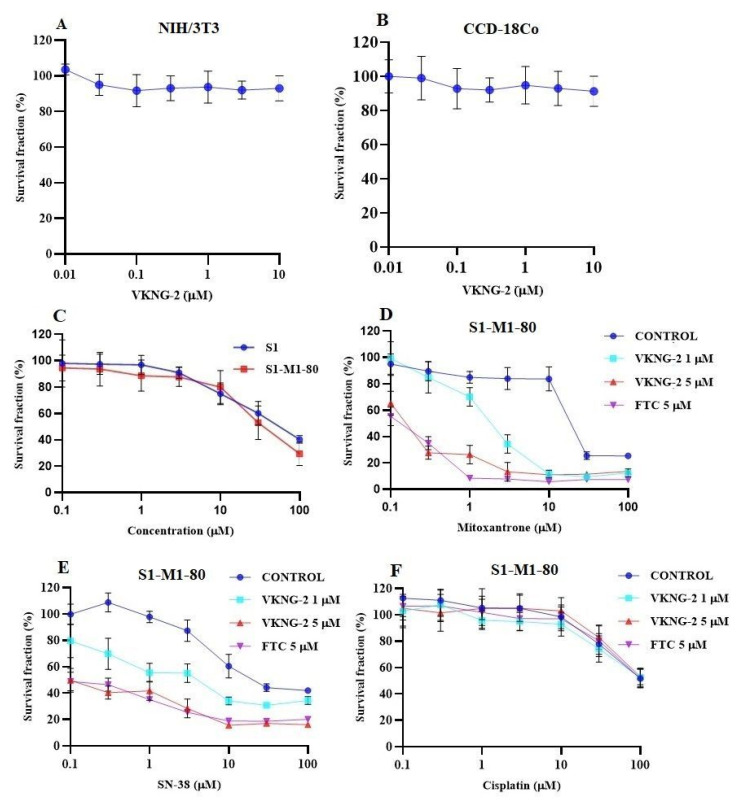
The effect of VKNG-2 in transformed mouse fibroblast (NIH/3T3) and human colon fibroblast (CCD-18Co) cell lines (**A**,**B**). The effect of VKNG-2 in S1 parental and ABCG2-overexpressing S1-M1-80 colon cancer cells (**C**). The survival fraction (%) was determined following incubation with 5 µM of VKNG-2 for 72 h in S1 (blue) and S1-M1-80 (red) cell lines and IC_50_ values of mitoxantrone (**D**), SN-38 (**E**), and cisplatin (**F**) in parental S1 and drug-selected ABCG2 overexpressing S1-M1-80 colon cancer cells with or without VKNG-2. The points with error bars represent the mean ± SD of independent determinations in triplicate. The figures are representative of three independent experiments.

**Figure 2:**
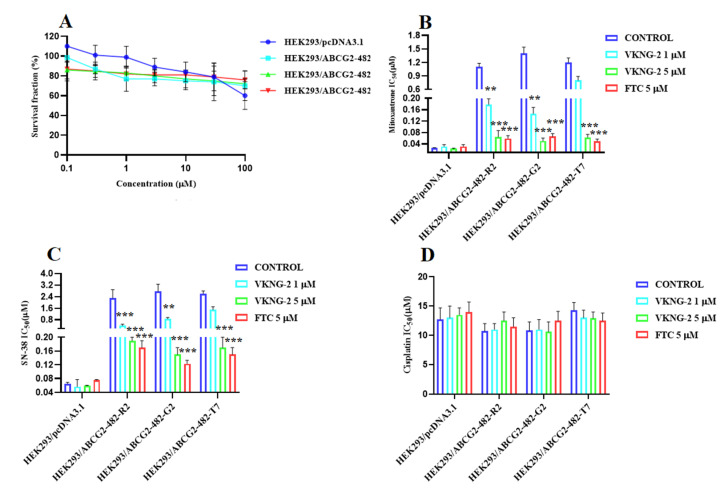
The effect of VKNG-2 in HEK293 cells transfected the gene coding for the ABCG2 transporter. (**A**) The survival fraction (%) for HEK293/pcDNA3.1 (empty DNA vector control), HEK293/ABCG2-482-R2, HEK293/ABCG2-482-G2, and HEK293/ABCG2-482-T7 cell lines was determined following incubation with 5 µM of VKNG-2 for 72 h. The IC50 values for mitoxantrone following incubation with VKNG-2 (1 or 5 µM) or FTC (5 µM) for 72 h in HEK293/pcDNA3.1 (empty DNA vector control), HEK293/ABCG2-482-R2 (HEK293/ABCG2-482-G2 and HEK293/ABCG2-482-T7 cell lines. The IC_50_ values of mitoxantrone (**B**), SN-38 (**C**), and cisplatin (**D**) in HEK293/pcDNA3.1 (empty DNA vector control), HEK293/ABCG2-482-R2, HEK293/ABCG2-482-G2, and HEK293/ABCG2-482-T7 cell lines. The points with error bars represent the mean ± SD of independent determinations in triplicate. The figures are representative of three independent experiments. ** *p* ≤ 0.01 and *** *p* < 0.001 compared to the control group.

**Figure 3:**
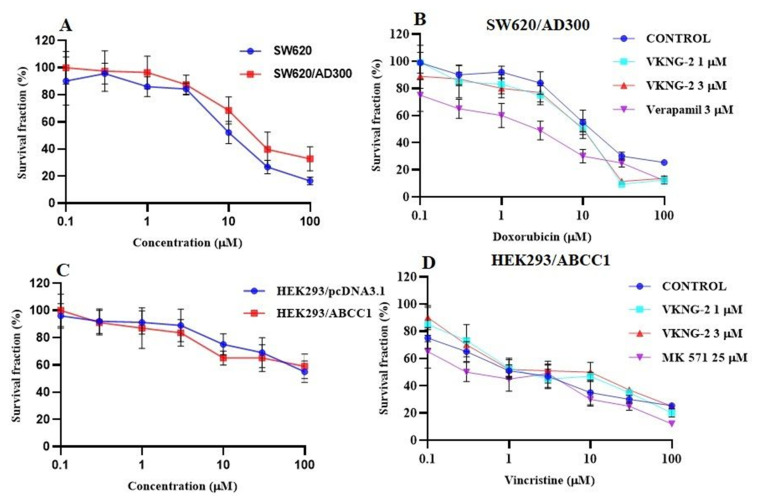
The effect of VKNG-2 in SW620 parental, ABCB1-overexpressing SW620/Ad300 colon cancer cells and HEK293/pcDNA3.1 parental and HEK293/ABCC1 transfected cells. (**A**) The survival fraction (%) for the SW620 parental and SW620/Ad300 colon cancer cell lines were determined following incubation with VKNG-2 for 72 h. (**B**) The IC50 values of doxorubicin in the presence of vehicle (Control), VKNG-2 (1 or 3 μM) or verapamil (3 μM) for 72 h in SW620 parental and SW620/Ad300 colon cancer cells. (**C**): The survival fraction (%) for the HEK293/pcDNA3.1 (empty DNA vector control) and HEK293/ABCC1 (transfected with the DNA coding for the ABCC1 transporter) cells were determined following incubation with VKNG-2 for 72 h. (**D**) The IC_50_ values of vincristine in the presence of vehicle (Control), VKNG-2 (1 or 3 μM) or MK-571 (25 μM) for 72 h in HEK293/pcDNA3.1 and HEK293/ABCC1 cells. The points with error bars represent the mean ± SD of independent determinations in triplicate. The figures are representative of three independent experiments.

VKNG-2 was chosen based on a previous study that reported the synthesis of benzamide compounds with either urea or an amide linker [37]. The transformed mouse fibroblast (NIH/3T3) and human colon fibroblast cells (CCD-18Co) were incubated with different concentrations of VKNG-2 (0–10 µM) for 72 h and the results indicated that more than 90% of cells survived even at a high concentration (10 µM) of VKNG-2 (Figure 1A,B). Furthermore, in the human fibroblast cell line, CCD-18Co, the transformed mouse fibroblast cell line, NIH/3T3 and the human embryonic kidney cell line, the percentage of viable cells after incubation with 1 or 5 μM of VKNG-2, was 94 and 93%, 93 and 92% and 91 and 85%, respectively. Based on the cytotoxicity data, the concentrations at which approximately 85% of parental and resistant cells survived (1 and 5 µM of VKNG-2) were selected for the reversal experiments. (Figure 1C and Figure 2A). These concentrations of VKNG-2 significantly increased the cytotoxicity of mitoxantrone (Figure 1D) and SN-38 in the S1-M1-80 (Figure 1E) and the transfected resistant cancer cell lines, HEK293/R482, HEK293/R482G, and HEK293/R482T to mitoxantrone (Figure 2B) and SN-38 (Figure 2C). The drug selected cell lines have characteristics similar to those *in vivo* but multiple factors that can cause MDR other than the overexpression of the ABCG2 transporter [38]. Furthermore, the role of ABCG2-mediated resistance can be confirmed using transfected cell lines, although these cells are non-cancerous. In the presence of 5 µM of VKNG-2, the resistance-fold values were reversed in S1-M1-80 colon cancer cells incubated with mitoxantrone (from 72- to 0.6–fold resistant) and SN-38 (114- to 0.5–fold resistant) individually (Table 2). Moreover, 5 µM of VKNG-2 reversed the resistance to mitoxantrone and SN-38 in the ABCG2 transfected cell lines (Table 3). The reversal efficacy of 5 μM of VKNG-2 for the ABCG2 transporter was comparable to that of the same concentration of FTC (a known ABCG2 inhibitor). Furthermore, VKNG-2, compared to vehicle, did not significantly alter the efficacy of these substrate drugs in the S1 parental non-resistant cell line. VKNG-2, at 5 µM, did not significantly alter the efficacy of cisplatin, which is an anticancer drug that is not a substrate for the ABCG2 transporter [39] (Figure 1F and Figure 2D). Finally, 3 µM of VKNG-2 did not significantly reverse the resistance of (1) SW620/AD300 cancer cells, which overexpress ABCB1 transporter, to doxorubicin (Figure 3B), (2) HEK 293/ABCC1 cells, overexpressing the ABCC1 transporter, to vincristine (Figure 3D). Overall, our results suggest that VKNG-2 reverses the resistance to mitoxantrone and SN-38 in wild-type and mutant-type cancer cell lines that overexpress the ABCG2 transporter.

### 2.2. VKNG-2 Significantly Decreases the Efflux of [^3^H]-mitoxantrone in S1-M1-80 Colon Cancer Cells 

**Figure 4:**
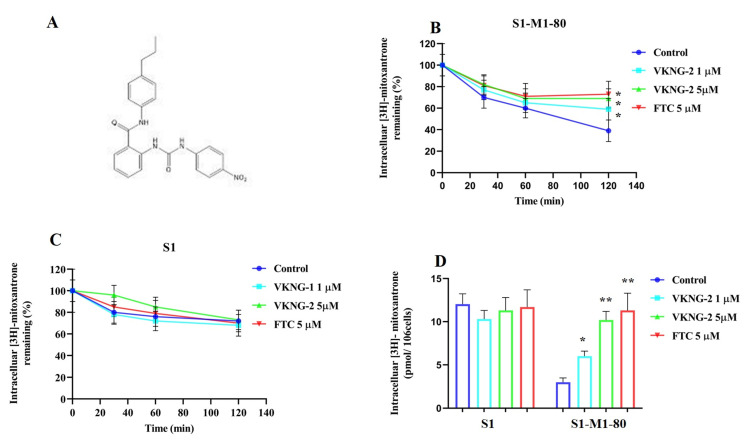
(**A**) The chemical structure of VKNG-2. (**B**) The effect of the incubation of vehicle (Control), VKNG-2 (1 or 5 µM) or FTC (5 µM) for 30, 60 or 120 min on the efflux of the ABCG2 transporter substrate, [^3^H]-mitoxantrone from S1-M1-80 colon cancer cells overexpressing the ABCG2 transporter. (**C**) The effect of the incubation of vehicle (Control), VKNG-2 (1 or 5 µM) or FTC (5 µM) for 30, 60 or 120 min on the efflux of the ABCG2 transporter substrate, [^3^H]-mitoxantrone from S1 parental colon cancer cells. (**D**) The effect of the vehicle (Control), VKNG-2 (1 or 5 µM) or FTC (5 µM) on the intracellular accumulation of [^3^H]-mitoxantrone in S1 and S1-M1-80 colon cancer cells. The columns are the mean of triplicate determinations; the error bars represent the SD. * *p* ≤ 0.05 and ** *p* ≤ 0.01 compared with the control group.

To determine the mechanism by which VKNG-2 reverses drug resistance, we measured the accumulation and efflux of [^3^H]-mitoxantrone in the parental cell line, S1, and its mitoxantrone resistant colon cancer cell line, S1-M1-80. The accumulation of [^3^H]-mitoxantrone in the mitoxantrone-resistant S1-M1-80 colon cancer cells was significantly lower than that of the parental S1 colon cancer cells (Figure 4B). However, the incubation of S1-M1-80 colon cancer cells with 5 μM of VKNG-2 significantly increased the intracellular accumulation of [^3^H]-mitoxantrone compared to cells incubated with vehicle (Figure 4D). In contrast, 5 µM of VKNG-2 treatment did not significantly alter the accumulation of [^3^H]-mitoxantrone in the parental S1 colon cancer cell line compared to the vehicle as these cells do not overexpress the ABCG2 transporter and thus are not resistant to mitoxantrone. These results suggest that VKNG-2 increases the level of [^3^H]-mitoxantrone in S1-M1-80 cells. 

To determine if the increase in the accumulation of [^3^H]-mitoxantrone in MDR cells was due to VKNG-2 preventing the ABCG2 transporter efflux activity, we measured [^3^H]-mitoxantrone levels in the drug-resistant cells in the presence or absence of VKNG-2. In the S1-M1-80 colon cancer cells, the intracellular levels of [^3^H]-mitoxantrone were 70%, 60%, and 39% that of the S1 parental colon cancer cell line, at 30, 60, and 120 min, respectively, in the absence of VKNG-2 (Figure 4B), suggesting that the efflux of [^3^H]-mitoxantrone was mediated by the ABCG2 transporter. In contrast, 5 µM of VKNG-2 substantially decreased the efflux of [^3^H]-mitoxantrone compared to cells incubated with vehicle, thereby increasing the intracellular levels of [^3^H]-mitoxantrone (82% at 30 min, 69% at 60 min, and 69% at 120 min; (Figure 4B) The magnitude of the decrease in the efflux of [^3^H]-mitoxantrone by VKNG-2 was comparable to that of 5 µM of FTC. The intracellular levels of [^3^H]-mitoxantrone in the S1 parental cancer cells were not significantly altered by 5 µM of VKNG-2 in comparison with the cells treated with vehicle. (Figure 4C). 

Overall, our results indicated that VKNG-2 significantly increased the accumulation of [^3^H]-mitoxantrone in S1-M1-80 cancer cells resistant to mitoxantrone by blocking the efflux function of the ABCG2 transporter. 

### 2.3. The Effect of VKNG-2 on the Expression of the ABCG2 Transporter

**Figure 5:**
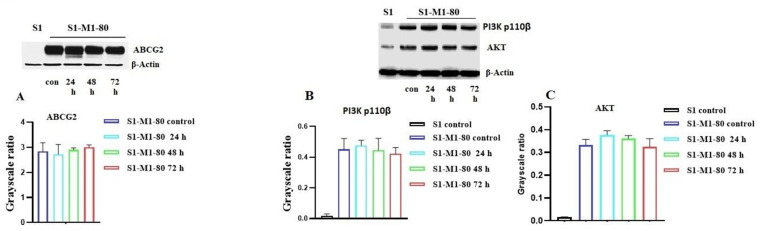
The effect of VKNG-2 on the expression of the ABCG2, PI3K p110β and AKT protein. The effect of VKNG-2 on the expression of the ABCG2, PI3K p110β and AKT protein were determined in S1-M1-80 colon cancer cells following incubation with vehicle (Control) or 5 μM of VKNG-2 for 24, 48 or 72 h (**A**–**C**). Equal amounts of total cell lysates were used for each sample and a Western blot analysis was performed.

The reversal of the ABCG2-mediated MDR by VKNG-2 could result from either downregulation of the expression level of the ABCG2 transporter and/or by inhibiting the efflux function of ABCG2. Furthermore, it has been reported that the activation of the PI3K/AKT pathway increases the expression of the ABCG2 protein, thereby increasing the likelihood of MDR in certain cancer cells [40,41]. Therefore, we used Western blotting to assess the effect of VKNG-2 on the ABCG2, PI3K- p110β and AKT protein expression in S1-M1-80 cancer cells. The incubation of S1-M1-80 cancer cells with 5 µM of VKNG-2 for 72 h did not significantly alter the expression level of the ABCG2 (Figure 5A), PI3K p110β (B) and AKT (C) proteins compared to cells incubated with the vehicle. These results, in tandem with the [^3^H]-mitoxantrone efflux results, indicate that the reversal efficacy of VKNG-2 in S1-M1-80 cancer cells was due to its inhibition of the ABCG2 efflux function. 

### 2.4. The Effect of VKNG-2 on the ATPase Activity of the ABCG2 and ABCB1 Transporter 

**Figure 6:**
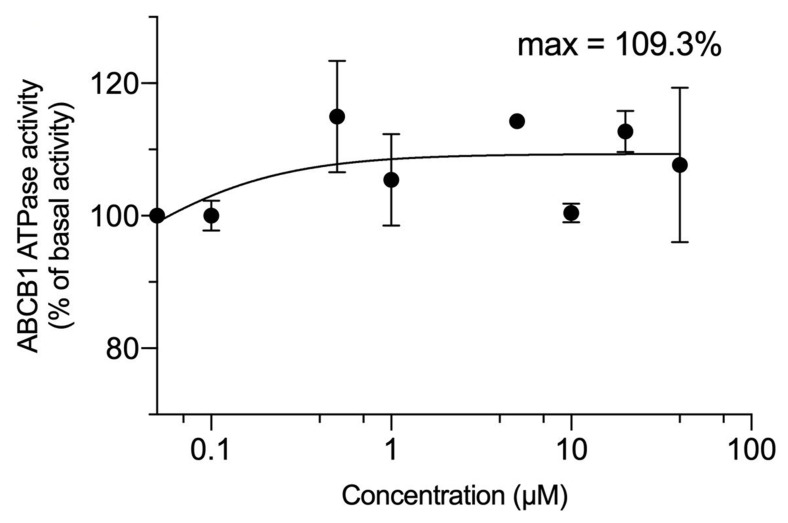
VKNG-2 stimulated the ATPase activity of ABCB1. The graph illustrates the effect of 0–100 µM of VKNG-2 on the ATPase activity of ABCB1.

The drug efflux function of the ABCG2 transporter has been reported to be linked to ATP hydrolysis and it can be stimulated or inhibited by ABCG2 substrates [42]. The stimulation of ABCG2 transporter ATPase activity suggests that the test compound interacts with the transporter at the drug-substrate-binding site [43]. Our results indicated that VKNG-2 produced a maximal increase that was 3.5-fold greater than the basal activity, with an EC_50_ value of 2.3 µM [37]. The stimulation of ABCG2 ATPase activity by VKNG-2 suggests that it could interact with the drug-ABCG2 substrate binding site. VKNG-2 produced a 1-fold increase in the basal activity of the ABCB1 ATPase. (Figure 6).

### 2.5. The Effect of VKNG-2 on the Cellular Localization of the ABCG2 Transporter Protein 

**Figure 7:**
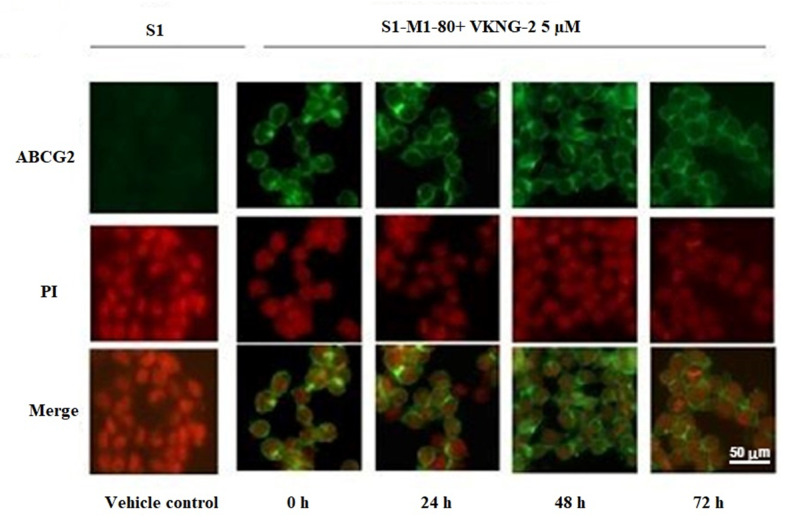
The effect of VKNG-2 on the expression and localization of ABCG2 using immunofluorescence. The effect of the incubation of S1 and S1-M1-80 colon cancer cells with vehicle (Control) or 5 µM of VKNG-2 for incubated for 0, 24, 48 or 72 h. The green color represents the presence of the ABCG2 transporter, and the red color represents the nucleus.

The reversal of mitoxantrone resistance in the S1-M1-80 drug-resistant cancer cells by VKNG-2 could also result from an alteration in the subcellular localization of ABCG2 from the cell membrane (i.e., the transporter would not be in the cell membrane, producing a decrease in drug efflux from the cells). Thus, we used an immunofluorescence assay to determine if VKNG-2 alters the localization of the ABCG2 transporter protein from the cell surface to the cytoplasm. The incubation of S1-M1-80 cells with 5 µM of VKNG-2 for 72 h did not result in the subcellular localization of the ABCG2 transporter from the cell membrane to the cytosol. (Figure 7).

### 2.6. The Docking Simulation of VKNG-2 in the Drug-binding Pocket of the Human ABCG2, ABCB1 and ABCC1 Protein

**Figure 8:**
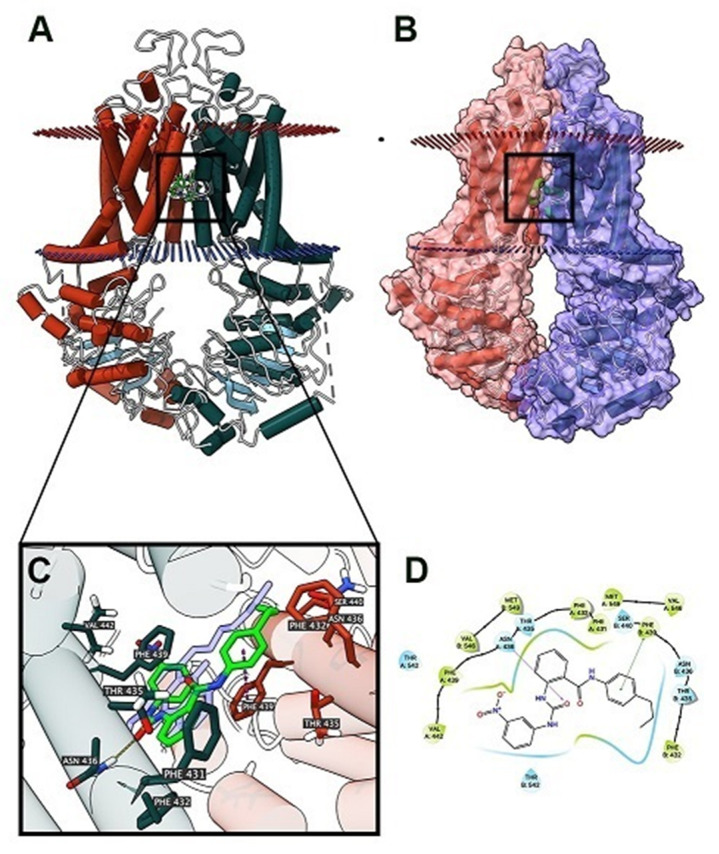
The molecular modeling of VKNG-2 and human ABCG2. (**A**) An overview of mitoxantrone and the best-scoring pose of VKNG-2 in the drug-substrate binding site of the ABCG2 protein (6VXI). The cytoplasm membrane is depicted as dotted planes, where the red or blue planes indicate the extracellular or intracellular side, respectively. ABCG2 is displayed as colored tubes and ribbons. VKNG-2 and mitoxantrone are displayed as colored sticks. Carbon: lime green (VKNG-2) or white (mitoxantrone); oxygen: red; nitrogen: blue. (**B**) Mitoxantrone and the best-scoring pose of VKNG-2 in the drug-substrate binding site of the ABCG2 protein with molecule surface displayed. (**C**) Details of the interactions between VKNG-2 and the ABCG2 (6VXI) drug-substrate binding site. ABCG2 protein helices are displayed as colored tubes (chain A: green; chain B: red). Important residues are displayed as colored sticks (carbon: same as chain color; oxygen: red; nitrogen: blue). VKNG-2 is displayed as colored sticks (carbon: lime; oxygen: red; nitrogen: blue). Hydrogen bonds are displayed as yellow dash lines. p-p stacking interactions are displayed as magenta dash lines. (**D**) 2D diagram of the interaction between VKNG-2 and ABCG2. Important amino acids are displayed as colored bubbles (green: hydrophobic; blue: polar). Purple solid lines with an arrow indicate hydrogen bonds. Green solid lines without arrow indicate p-p stacking interactions.

**Figure 9:**
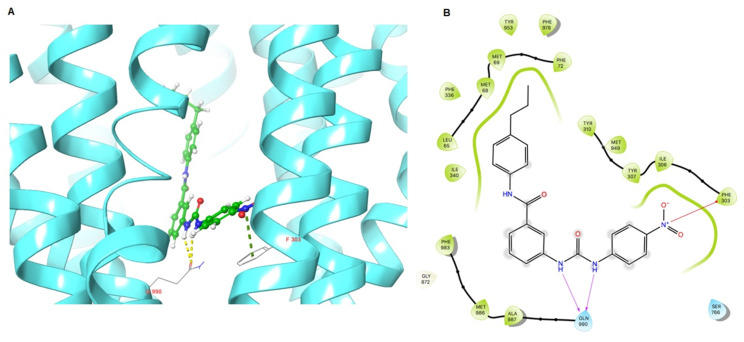
Molecular interaction of VKNG-2 with the human ABCB1 model. (**A**) Docking pose of VKNG-2 within the binding pocket of ABCB1. The protein is represented as sky blue colored ribbons. Amino acid residues are represented as follows: carbon in gray, hydrogen in white, nitrogen in blue and oxygen in red. The ligand is represented by the ball and stick model with carbon atoms represented in green, oxygen in red nitrogen in blue and hydrogen in white. Green dashes represent π-cation interactions and yellow dashes represent hydrogen bonding. (**B**) 2-D ligand interaction between VKNG-2 and ABCB1. Red arrows indicate π-cation interaction with amino acid residues within 5 Å of the ligand and the magenta arrows represent hydrogen bonding.

As VKNG-2 inhibited the efflux function of the ABCG2 transporter, we conducted a docking simulation with mitoxantrone (an ABCG2 substrate) at the binding site (6VXI) of the ABCG2 transporter. Our results indicated that VKNG-2 docked into the substrate-binding site, with a docking score of −10.2 kcal/mol, which is shown in Figure 8. The binding of VKNG-2 to the ABCG2 protein was primarily due to hydrophobic interactions. VKNG-2 is located and stabilized in the hydrophobic cavity formed by Phe431, Phe432, Phe439, Val442, Val546, Met549 in chain A and Phe432, Phe439, Val546, Met 549 in chain B. Furthermore, the binding of VKNG-2 was stabilized by a hydrogen bond formed with Asn436 in chain A and a π-π stacking with Phe439 in chain B. Based on the docking images, π-π interactions were formed between the phenyl of the Phe439 and phenyl group adjacent to the alkyl chain of VKNG-2 and a hydrogen bond interaction between the carbonyl group of the urea in VKNG-2 and the amino hydrogen of Asn436. In the 6QEX structure (ABCB1), a hydrogen bond was formed between the carbonyl of the side chain of Gln 990 and the nitrogen of VKNG-2 and there was a π-bond interaction between the phenyl side chain of Phe 303 and the nitro group of VKNG-2 (Figure 9). The docking score of VKNG-2 for the ABCB1 transporter was −6.660 kcal/mol (Figure 9).

**Figure 10:**
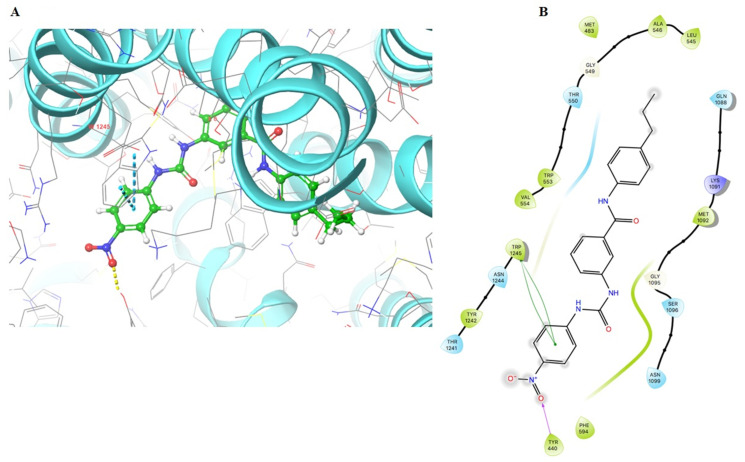
Molecular interaction of VKNG-2 with the human ABCC1 model. (**A**) Docking pose of VKNG-2 within the binding pocket of ABCC1. The protein is represented as sky blue colored ribbons. Amino acid residues are represented as follows: carbon in gray, hydrogen in white, nitrogen in blue and oxygen in red. The ligand is represented by the ball and stick model with carbon atoms represented in green, oxygen in red nitrogen in blue and hydrogen in white. Blue dashes represent π-π stacking interaction, yellow dashes represent the hydrogen bonding. (**B**) 2-D ligand interaction between VKNG-2 and ABCC1. Green indicates π-π interaction with amino acid residues within 5 Å of the ligand and the magenta arrow represents hydrogen bonding.

The docking results for the ABCC1 transporter indicated a double π-π interaction between the para-nitrophenyl ring and the indole ring of the Trp1245 and a hydrogen bond formed with the O_2_ on the nitro group and the hydroxyl on the side chain of Tyr440. The docking score of VKNG-2 for the ABCC1 transporter was −6.1 kcal/mol (Figure 10).

## 3. Discussion and Conclusions

Previously, we reported the design, synthesis and pharmacological evaluation of novel benzamide analogs derived from tariquidar, a third-generation selective inhibitor of P-gp, in cancer cells overexpressing the ABCG2 transporter [37]. The benzamide analogs had selectivity for inhibiting the ABCG2 transporter compared to the ABCB1 transporter. One of the benzamide analogs, VKNG-2, containing a urea linker, was the most efficacious in inhibiting the ABCG2 transporter in MX20 and S1-M1-80 resistant cancer cells. Therefore, experiments were performed to determine (1) the efficacy of VKNG-2 to reverse the ABCG2-mediated resistance to mitoxantrone, SN-38 and cisplatin in S1-M1-80 colon cancer cells and (2) the mechanisms by which VKNG-2 reverses MDR in S1-M1-80 colon cancer cells. Based on the survival fraction experiments, we used 1 and 5 μM of VKNG-2 for all experiments as these concentrations did not decrease the viability of either the S1 parental or S1-M1-80 colon cancer cells below 85% after 72 h of incubation. As previously shown, the *in vitro* efficacy of mitoxantrone and SN-38 were significantly decreased in S1-M1-80 colon cancer cells, compared to the parental S1 colon cancer cells [44].

VKNG-2 did not significantly alter the anticancer efficacy of mitoxantrone or SN-38 in the parental S1 colon cancer cells that do not overexpress the ABCG2 transporter. This finding is congruent with previous studies reporting that the efficacy of certain anticancer drugs in parental cancer cell lines that do not express the ABCG2 transporter is not altered by compounds that decrease the efflux and/or expression of the ABCG2 transporter [45,46,47]. However, in the S1-M1-80 colon cancer cells, 1 or 5 µM of VKNG-2 significantly increased the efficacy (i.e., decreased resistance) of the ABCG2 substrates, mitoxantrone and SN-38, in comparison with the cells incubated with the vehicle similar to FTC, as previously reported [48], suggesting that one of the mechanisms by which VKNG-2 reverses ABCG2-mediated drug resistance is by inhibiting the ABCG2 transporter. VKNG-2did not significantly alter the efficacy of cisplatin in S1-M1-80 or S1 parental colon cancer cells [49]. This result is consistent with previous studies indicating that cisplatin is not an ABCG2 specific substrate [39] and thus, its efficacy would not be altered by inhibiting the ABCG2 transporter. The cytotoxicity results in human and transformed mouse fibroblast cells indicated that the survival percentage was significantly higher for the non-cancer cells than the cancer cells, suggesting that VKNG-2 preferentially targeted the cancer cells and that it has a more acceptable cellular toxicity profile compared to other chemotherapeutic drugs that are ABCG2 substrates. 

In the wild-type ABCG2 protein, the amino acid arginine is located at position 482 on the carboxy terminus of the third transmembrane segment of the membrane-spanning domain, where substrate and drug binding occur [50]. Previously, it has been reported that resistance to mitoxantrone, doxorubicin, daunorubicin and SN-38 occurs in the cell containing the substitution of T or G for arginine at position, 482. Our findings are congruent with studies reporting that the R482G and R482T mutations in HEK293 transfected cells produce resistance to mitoxantrone and SN-38 [51]. VKNG-2, at 1 or 5 µM, and FTC, at 5 µM, significantly increased the efficacy of mitoxantrone and SN-38 in HEK293R2/G2 and T7 cells. Given that the overexpression of the ABCG2 gene in the transfected HEK293 cells is likely the only mechanism that mediates the resistance to mitoxantrone and SN-38, the results suggest that VKNG-2’s efficacy is due primarily to its interaction with the ABCG2 transporter. In S1-M1-80 colon cancer cells, neither FTC nor VKNG-2 significantly alters the efficacy of cisplatin compared to the control.

VKNG-2, at 1 or 3 μM, did not significantly alter the efficacy of vincristine and doxorubicin in HEK293/ABCC1 and SW620/Ad300 colon cancer cells which overexpress the ABCC1 and ABCB1 transporter. Overall, our results suggest that VKNG-2 is interacting with the ABCG2 transporter, thereby increasing the efficacy (i.e., reverses MDR) of the ABCG2 substrates, mitoxantrone and SN-38, in the MDR S1-M1-80 colon cancer cells. Based on our findings indicating that VKNG-2 selectively inhibits the ABCG2 transporter, we conducted experiments to determine the mechanism by which VKNG-2 reverses the resistance to mitoxantrone and SN-38.

We subsequently determined the effect of VKNG-2 on the accumulation of the ABCG2 substrate, [^3^H]-mitoxantrone, in S1-M1-80 colon cancer cells. Given that VKNG-2 does not increase the uptake of [^3^H]-mitoxantrone in colon cancer cells, our results suggest that VKNG-2 could increase the intracellular accumulation of [^3^H]-mitoxantrone by inhibiting the efflux activity of the ABCG2 transporter. However, it is possible that VKNG-2 could increase the efficacy of certain anticancer drugs that are ABCG2 substrates by decreasing the expression of the ABCG2 protein. 

Based on the ^3^H-mitoxantrone results, Western blotting assays were conducted to determine the effect of VKNG-2 on the *in vitro* expression of the ABCG2 transporter protein in S1-M1-80 colon cancer cells. The results indicated that the incubation of S1-M1-80 cells with VKNG-2- did not significantly alter the expression of the ABCG2 protein compared to control. Therefore, in this study, it is unlikely that the increase in the efficacy of mitoxantrone and SN-38 in S1-M1-80 colon cancer cells results from a decrease in the expression of the ABCG2 transporter protein. Previously, it has been shown that catalytic subunits of phosphatidylinositol 3-kinase (PI3K) and AKT are involved in mediating ABC transporter MDR. Therefore, we conducted experiments to determine if VKNG-2 decreases the expression of PI3K-p110β and AKT. However, VKNG-2, at 5 µM, did not significantly alter the expression of PI3K p110β and AKT in the MDR resistant cell line.

However, it is possible that an incubation period > 72 h with VKNG-2 could significantly alter ABCG2 protein expression, although additional experiments must be conducted using longer incubation times to validate this possibility. It is also possible that VKNG-2 could affect the localization of the ABCG2 protein from the cell membrane, thus decreasing the number of ABCG2 transporter available for drug efflux. Consequently, we performed *in vitro* immunofluorescent experiments to determine the subcellular localization of the ABCG2 protein. The results indicated that 5 µM of VKNG-2 is unlikely to increase the efficacy of mitoxantrone or SN-38 by altering the cellular localization of the ABCG2 transporter. Again, as with the ABCG2 protein expression experiments, we cannot rule out the possibility that longer incubation times may have significantly alter the cellular distribution of the ABCG2 transporter.

As previously mentioned, the efflux function of the ABCG2 transporter is coupled to ATP hydrolysis, which is increased by ABCG2 substrates, including mitoxantrone and SN-38. Therefore, we assessed the *in vitro* effect of VKNG-2 on the hydrolysis of ATP by the ATPase domain of the ABCG2 transporter. Our results indicated that VKNG-2 produced a concentration-dependent increase in ATPase activity, with a maximal stimulation that was 3.5-fold greater than the basal activity. The VKNG-2-induced increase in ABCG2 ATPase activity could be due to the competitive inhibition of the efflux of ABCG2 transporter substrates by interacting with the drug-substrate binding site present in the transmembrane domain. VKNG-2 produced a relatively small increase in the activity of the ABCB1 ATPase, suggesting that VKNG-2 does not significantly interact with the substrate-binding site on the ABCB1 transporter, a finding that is consistent with the docking study data for the ABCB1 and ABCC1 transporters. Thus, the docking and ATPase data support the concept that VKNG-2 selectively reverses MDR mediated by the ABCG2 transporter. The determination of the effect of VKNG-2 on the binding of radioligands that interact with the ABCG2 transporter substrate-drug binding site would be useful in determining if VKNG-2 directly interacts with this domain. 

To gain additional insight into the mechanism of action for VKNG-2, we performed molecular docking analysis to assess the interaction of VKNG-2 with the ABCG2 transporter, using a human ABCG2 model. The docking score for VKNG-2 was −10.2 kcal/mol and VKNG-2 is stabilized primarily by hydrophobic interactions and one π-π interaction and a hydrogen bond. Thus, our docking data indicates that VKNG-2 interacts with the drug-substrate binding pocket in the human homology model of the ABCG2 transporter. Consequently, it can be ascertained that VKNG-2 binds to the ABCG2 receptor and prevents the binding of other substrates to the same receptor. This interaction would inhibit the efflux of substrates such as mitoxantrone, thus increasing the efficacy of mitoxantrone in ABCG2 resistant cancer cells. Finally, VKNG-2 has a low docking score for the ABCB1 transporter (−6.6 kcal/mol) and a docking score of −6.1 kcal/mol for the ABCC1 transporter. Therefore, based on these results, we conclude that VKNG-2 has the strongest interaction with the ABCG2 transporter. 

Previous studies have reported that the overexpression of the ABCG2 transporter mediates MDR in different types of cancer, such as breast cancer, NSCLC (non-small cell lung cancer), ovarian cancer, chronic myeloid leukemia and gastric cancers [52,53,54,55]. Therapies targeting the ABCG2 transporter have been considered to be one of the emerging strategies to overcome drug resistance in cancer patients and modulating ABCG2-mediated efflux of chemotherapeutic drugs by ABCG2 reversal compounds has been reported to be efficacious in *in vitro* and *in vivo* models [56,57]. Furthermore, a series of extracellular-related signal kinases (ERK) specific inhibitors, tyrosine kinase inhibitors, and VEGFR inhibitors such as ulixertinib, dacomitinib and ZM323881, have been used as chemosensitizers to circumvent ABCG2-regulated MDR [58,59,60]. We hypothesize that VKNG-2 should be efficacious in reversing ABCG2-mediated chemotherapeutic resistance in NSCLC, ovarian and gastric cancers as these cancers overexpress ABCG2 transporter, although this remains to be confirmed.

In conclusion, VKNG-2 reverses ABCG2-mediated MDR by inhibiting the efflux function of the ABCG2 transporter, thereby increasing the intracellular concentrations of substrate chemotherapeutic drugs. Also, our results indicate that the reversal of MDR is not due to the downregulation of the expression of the ABCG2 protein or the alteration of the subcellular localization of the ABCG2 transporter. Finally, it is important to note that the *in vitro* results are insufficient for determining the potential clinical use of a drug and therefore, further data must be obtained using *in vivo* animal models to determine the efficacy and safety of VKNG-2.

## 4. Materials and Methods 

### 4.1. Chemicals

VKNG-2 (2-(3-(3-Nitrophenyl) ureido)-N-(4-propylphenyl)benzamide) (Figure 4A) was synthesized as previously described [37]. Mitoxantrone was bought from Enzo Sciences (Farmingdale, NY, USA) and doxorubicin was acquired from Medkoo Biosciences (Morrisville, NC, USA). SN-38, cisplatin, verapamil, vincristine, 3-(4,5-dimethylthiazol-yl)-2,5-diphenyltetrazolium bromide (MTT), and dimethyl sulfoxide (DMSO) were procured from Sigma Chemical (St. Louis, MO, USA). Fumitremorgin C (FTC) was obtained as a gracious donation from the Thomas McCloud Laboratory, NIH (Bethesda, MD, USA). Dulbecco’s modified Eagle’s medium (DMEM), 0.25% trypsin, penicillin/streptomycin (P/S), and fetal bovine serum (FBS) were obtained from Hyclone (Waltham, MA, USA). [^3^H]-mitoxantrone (4 Ci/mmol) was purchased from Moravek Biochemicals, Inc (Brea, CA, USA). Monoclonal antibodies D5V2K (selective against ABCG2), D6A8 (against β-Actin), C33D4 (against PI3K p110β), 9272 (selective against AKT) and secondary anti-rabbit antibody linked with HRP were obtained from Cell Signaling (Danvers, MA, USA). BXP-21 (specific against ABCG2) and Alexa fluor conjugated secondary antibody were obtained from Molecular Probes (Eugene, OR, USA). 

### 4.2. Equipment

The AccuSkan GO microplate reader was obtained from Thermo fisher (Fisher Sci., Fair Lawn, NJ, USA) and the TRI-CARB1 1900CA liquid scintillation analyzer was obtained from Packard Instrument Company, Inc (Downers Grove, IL, USA).

### 4.3. Cell Lines

The human colon cancer parental cell line, S1, mitoxantrone-selected ABCG2 overexpressing drug-resistant S1-M1-80 colon cancer cells, HEK293/pcDNA3.1 (human embryonic kidney cell line transfected with empty vector), HEK293 cells those are transfected with the ABCG2 transporter DNA, HEK293/R482 (wild-type) and HEK293/R482G and HEK293/R482T (2 variants), SW620 (parental) and SW620/AD300 colon cancer cells (ABCB1 overexpressing drug-resistant), transformed mouse fibroblast cell line, NIH/3T3 were cultured in DMEM medium and 10% FBS and 1% penicillin and streptomycin(PS). The human colon fibroblast cell line, CCD-18Co, was grown in EMEM medium with 10%FBS and 1% PS. All cells were incubated in 5% CO_2_ at 37 °C. The S1-M1-80 mitoxantrone-resistant colon cancer cells were grown in the presence of the anticancer drug, mitoxantrone (80 µM), which induced the overexpression of the ABCG2 transporter [61,62]. HEK293 cell lines were grown by selecting them with G418 (Geneticin, an aminoglycoside antibiotic), at a concentration of 2000 µg/mL, after transfecting HEK293 cells with an empty pcDNA3.1 vector or pcDNA3.1 vector containing the DNA for ABCG2 with containing wither an arginine (R), glycine (G) or threonine (T) at position 482 [63]. The HEK/pcDNA3.1 cell line was transfected with the DNA coding for the ABCC1 transporter to generate the HEK293/ABCC1 cell line [64,65]. SW620 and SW620/AD300 cells, S1 and ABCG2-overexpressing S1-M1-80 cells were kindly provided by Drs. Susan Bates (Columbia University, NY, USA) and HEK/pcDNA3.1, HEK293/R482, HEK293/R482G and HEK293R482T were obtained from Robert Robey (NCI, NIH, Bethesda, MD, USA). HEK293/ABCB1 was kindly provided by Dr. Suresh V. Ambudkar (NCI, NIH, Bethesda, MD, USA). The fibroblast cell lines, NIH/3T3 and CCD-18Co were purchased from ATCC (Manassas, VA, USA). 

### 4.4. MTT Assay for Cytotoxicity Determination and the Reversal Experiments

The MTT assay was performed in a 96 well plate to determine the concentration of VKNG-2 to be used in experiments with the parental and drug-resistant cell lines. A seeding density of 3 × 10^3^cells/well in 180 µL of the medium was used for the entirety of cell lines. VKNG-2 (0–100 µM) was added after the cells were attached. After 72 h of incubation, MTT solution (4 mg/mL) was added to each well and the cells were incubated for an additional 4 h at 37 °C. Subsequently, the supernatant was discarded and 100 μL of DMSO was added to each well to dissolve the formazan crystals. The absorbance was obtained using a spectrophotometer, which was set at 570 nm as previously described [66,67,68]. The concentrations at which around 85% of parental and resistant cells survived (i.e., 1 and 5 µM) were used for the reversal experiments. 

For the reversal experiments, S1 and S1-M1-80 colon cancer cells were cultured with 1 and 5 µM of VKNG-2, 5 µM of FTC, (ABCG2 transporter) [48], 3 µM of verapamil, (ABCB1 inhibitor) [69] or 25 µM of MK571, an inhibitor of the ABCC1 transporter [7] for 2 h. Following incubation, the chemotherapeutic drugs, (mitoxantrone, SN-38 and cisplatin), were added at different concentrations (20 µl/well) into the designated wells. The MTT assay was performed after 72 h of incubation by recording the absorbance at 570 nm.

### 4.5. [3. H]- Mitoxantrone accumulation and efflux assay

The intracellular accumulation of [^3^H]-mitoxantrone and the efflux activity of the ABCG2 transporter (at 0, 30, 60 and 120 min) in S1 parental and S1-M1-80 resistant cancer cells was determined in the presence or absence of 1 and 5 µM of VKNG-2 or 5 µM of FTC, as previously described [7]. Briefly, the cells were trypsinized and incubated in DMEM with or without the reversal compounds (VKNG-2 at 1 and 5 µM and FTC at 5 µM) at 37 °C for 2 h. Subsequently, the cells were incubated with 0.01 µM of [^3^H]-mitoxantrone with or without the inhibitor at 37 °C for an additional 2 h. The radioactivity was quantified using a scintillation counter. 

### 4.6. Western Blot Analysis

The expression of the ABCG2, PI3K - p110β and AKT proteins was determined using Western blotting as previously described [70]^.^ Briefly, the cell lysates were prepared from the parental S1 and drug-resistant S1-M1-80 cells after incubating with 5 µM of VKNG-2 for 24, 48 and 72 h. After protein quantitation using the BCA Protein Assay Kit (Thermo Scientific, Rockford, IL, USA), the protein samples were separated by PAGE and then transferred onto PVDF membranes. After blocking the membranes with 5% milk, the membranes were incubated with primary antibodies against ABCG2/PI3k p110β/AKT or β actin (1:1000) at 4 °C overnight, which was followed by further incubation with HRP-linked secondary antibody (1:1000) for 2 h at room temperature. The protein bands were visualized after exposed the membranes to Pierce™ ECL Western blotting substrate (Thermo Scientific, Rockford, IL, USA). The expression levels of the proteins were analyzed by ImageJ software. 

### 4.7. ATPase Assay

VKNG-2 induced vanadate-sensitive ATPase assay was performed as described previously [71]. In brief, ABCG2-overexpressing cell membranes purchased from BD Biosciences (San Jose, CA, USA) were incubated with an assay buffer containing 5 mM sodium azide (NaN_3_), 1 mM ouabain (g-strophanthin), 2 mM dithiothreitol (DTT), 10 mM magnesium chloride (MgCl_2_), 50 mM potassium chloride (KCl), 2 mM ethylene glycol-bis(β-aminoethyl ether)-N,N,N′,N′-tetra acetic acid (EGTA) and 50 mM pH 6.8 2-(N-morpholino) ethane sulfonic acid (MES), with or without 0.3 mM sodium orthovanadate (Na_3_VO_4_), at 37 °C for 5 min. The mixture was incubated with 0–100 µM of VKNG-2 at the same temperature for 3 min. The Mg-ATP (5 mM) solution was added to initiate a 20 min reaction at 37 °C, followed by adding 5% SDS to terminate the reaction. The amount of inorganic phosphates was determined by a colorimetric method, as previously described [72].

### 4.8. Immunofluorescence

To determine if the reversal efficacy of VKNG-2 is due to an alteration of the subcellular localization of membrane protein ABCG2, an immunofluorescence assay was performed [70]. For the immunofluorescence analysis, the parental (S1) and drug-resistant cells (S1-M1-80) were incubated with or without the vehicle or 5 µM of VKNG-2 for 24, 48 and 72 h. The cells were fixed with 4% formaldehyde, permeabilized by 0.25% Triton X-100, and blocked with 6% BSA followed by incubation with primary antibody against ABCG2 (1:1000). On the following day, the primary antibody was removed, and the cells were further incubated with Alexa Fluor 488 conjugated secondary antibody (1:1000) at room temperature for 2 h. Nuclei were stained using propidium iodide. Cell images were taken using a Nikon TE-2000S fluorescence microscope (Nikon Instruments Inc., Melville, NY, USA). The immunofluorescence images were taken, and all of the experiments were conducted independently in triplicate. 

### 4.9. Molecular Docking of VKNG-2 with the Human ABCG2, ABCB1 and ABCC1 Model

The three-dimensional structure of VKNG-2 was established for docking simulation using a previously reported human ABCG2 model. [73] The human ABCG2 protein model 6VXI (with mitoxantrone bound) was acquired from the Research Collaboratory for Structural Bioinformatics Protein Data Bank (RCSB PDB, http://rcsb.org, 29 July 2020). The 6VXI model which has mitoxantrone bound has only recently been reported. Both models represent an inward-facing human ABCG2 transporter protein, with a resolution of 3.7 Å [74]. The docking calculations were carried out using the program, AutoDock Vina (version 1.1.2) [75]. Hydrogen atoms and partial charges were attached using AutoDockTools (ADT, version 1.5.4). The docking grid center coordinates were estimated from the bound ligand mitoxantrone provided in the 6VXI PDB files. 

The docking studies were also performed for the ABCB1 (PDB 6QEX) and ABCC1(PDB 5UJA) transporters. Docking experiments were performed using a Mac Pro 6-core Intel Xenon E5 processor with a Macintosh Operating System (OS Sierra), using the Maestro v12. 3. 012 software. Ligand preparations for VKNG-2 were done using Lig-prep [76]. A homology model of human ABBC1 was imported from the protein data bank. Protein preparation of the homology model was performed using ‘Protein Preparation Wizard’. Extra precision docking was performed using a maximum of 10 poses [77].

The receptor/ligand preparation and docking simulation were achieved using the default settings. The top-scoring model, 6VXI (based on the docking score in kcal/mol) was chosen for additional investigation and visualization. 

### 4.10. Statistical Analysis

All experiments were performed at least three times and the data were analyzed using GraphPad Prism (version 8). The *a priori* significance level was *p* < 0.05 and the data were analyzed using a one-way or two-way ANOVA and *post hoc* analysis was conducted using Dunnett’s *post hoc* test. 

## Figures and Tables

**Table 1:** Mechanism of action and class of various drugs in CRC.

Drugs	Class	Target	References
5-fluorouracil	Fluoropyrimidine	Thymidylate synthase	[29]
Irinotecan	Camptothecin analogue	Topoisomerase I	[30]
Leucovorin	5-formyl derivative of tetrahydro folic acid	Decrease the toxic effects of methotrexate	[31]
Oxaliplatin	Platinum-based compound	Arrests DNA synthesis	[32]
Bevacizumab	Angiogenesis inhibitor	Anti-vascular endothelial growth factor (VEGF)	[33]
Cetuximab	Chimeric human- murine immunoglobulin G1 (IgG1) monoclonal antibody	Epidermal growth factor receptor (EGFR)	[34]
Anthracyclines	Cytostatic antibiotics	Generation of reactive oxygen species (ROS)	[35]
Methotrexate	Folic acid antagonist	Dihydrofolate reductase	[36]

**Table 2:** The effect of VKNG-2 on reversal of ABCG2 mediated MDR in the drug selective cell lines.

Cell Lines	S1		S1-M1-80	
Compounds	IC_50_ ± SD (µM)	FR	IC_50_ ± SD (µM)	FR
Mitoxantrone	0.31 ± 0.03	(1.0)	21.57 ± 3.23	[71.9]
+VKNG-2 (1 µM)	0.22 ± 0.03	(0.7)	2.13 ± 0.38	[7.1]
+VKNG-2 (5 µM)	0.25 ± 0.04	(0.7)	0.19 ± 0.04	[0.6]
+FTC (5 µM)	0.33 ± 0.03	(1.0)	0.15 ± 0.03	[0.5]
**SN-38**	0.22 ± 0.03	(1.0)	22.82 ± 3.95	[114.1]
+VKNG-2 (1 µM)	0.27 ± 0.05	(1.0)	4.73 ± 0.91	[23.5]
+VKNG-2 (5 µM)	0.19 ± 0.02	(1.0)	0.10 ± 0.01	[0.5]
+FTC (5 µM)	0.37 ± 0.06	(1.7)	0.09 ± 0.01	[0.5]
**Cisplatin**	99.37 ± 17.99	(1.0)	103.37 ± 14.37	[1.0]
+VKNG-2 (1 µM)	95.86 ± 10.55	(1.0)	107.13 ± 14.99	[1.0]
+VKNG-2 (5 µM)	115.27 ± 17.49	(1.3)	103.39 ± 12.51	[1.0]
+FTC (5 µM)	111.07 ± 17.33	(1.3)	109.37 ± 17.59	[1.1]

µM = Micromole, Values in tables are representative of at least three independent experiments performed in triplicates. IC_50_: concentration that inhibits cell survival by 50% (mean ± SD). FR: Resistance fold was calculated by dividing the _IC50_ values of substrates in the presence or absence of inhibitor by the _IC50_ of parental cells without inhibitor.

**Table 3:** The effect of VKNG-2 on reversal of ABCG2 mediated MDR in the transfected cell lines.

Cell Lines	HEK293/pcDNA3.1		HEK293/R2		HEK293/G2		HEK293/T7	
Compounds	IC_50_ ±SD (µM)	FR	IC_50_ ±SD (µM)	FR	IC_50_ ±SD (µM)	FR	IC_50_ ±SD (µM)	FR
Mitoxantrone	0.025 ± 0.002	[1.0]	1.181 ± 0.081	[47.3]	1.432± 0.139	[56.5]	1.233 ± 0.189	[49.3]
+ VKNG-2 (1µM)	0.031 ± 0.006	[1.25]	0.178 ± 0.023	[7.1]	0.146 ± 0.022	[5.1]	0.857 ± 0.009	[34.0]
+VKNG-2 (5 µM)	0.023 ± 0.002	[0.94]	0.064 ± 0.023	[2.5]	0.055 ± 0.010	[2.2]	0.067 ± 0.013	[2.5]
+FTC (5 µM)	0.031 ± 0.006	[1.26]	0.059± 0.01	[2.3]	0.066 ± 0.019	[2.7]	0.053 ± 0.007	[2.1]
**SN-38**	0.065± 0.005	[1.0]	2.379 ± 0.53	[35.3]	2.76 ± 0.52	[42.2]	2.665 ± 0.243	[34.3]
+VKNG-2 (1 µM)	0.057 ± 0.027	[0.8]	0.398 ± 0.157	[6.1]	0.86 ± 0.199	[13.0]	1.566 ± 0.277	[23.1]
+VKNG-2 (5 µM)	0.059 ± 0.003	[0.9]	0.198 ± 0.01	[3.0]	0.159 ± 0.020	[2.3]	0.173 ± 0.027	[2.7]
+FTC (5 µM)	0.075 ± 0.002	[1.0]	0.179 ± 0.02	[3.8]	0.123 ± 0.019	[1.9]	0.157 ± 0.035	[2.3]
**Cisplatin**	12.759 ± 2.33	[1.0]	10.56 ± 1.359	[0.9]	10.348 ± 1.579	[0.9]	14.323 ± 1.343	[1.1]
+VKNG-2 (1 µM)	13.985 ± 2.319	[1.1]	11.607 ± 1.464	[0.9]	11.997 ± 1.71	[1.1]	14.009 ± 1.317	[1.1]
+VKNG-2 (5 µM)	13.835 ± 1.295	[1.1]	12.156 ± 1.596	[1.0]	10.695 ± 1.913	[0.9]	12.964 ± 1.127	[1.0]
+FTC (5 µM)	14.950 ± 1.245	[1.1]	11.523 ± 1.525	[1.1]	12.541 ± 1.731	[1.0]	12.589 ± 1.311	[1.0]

µM = Micromole. Values in tables are representative of at least three independent experiments performed in triplicates. _IC50_: concentration that inhibits cell survival by 50% (mean ± SD). FR: Resistance fold was calculated by dividing the _IC50_ values of substrates in the presence or absence of inhibitor by the _IC50_ of parental cells without inhibitor.

## Data Availability

Not applicable.
